# Epstein–Barr virus expression in Hodgkin’s disease in relation to patient characteristics, serum factors and blood lymphocyte function

**DOI:** 10.1038/sj.bjc.6690827

**Published:** 1999-12

**Authors:** U Axdorph, A Porwit-MacDonald, J Sjöberg, G Grimfors, M Ekman, W Wang, P Biberfeld, M Björkholm

**Affiliations:** Department of Medicine, Division of Haematology Institutet; Department of Pathology and Cytology, Karolinska Hospital and Institute,SE-171 76 Stockholm, Sweden

**Keywords:** Epstein–Barr virus, Hodgkin’s disease, lymphocyte function, prognosis, sCD30, s-ICAM-1

## Abstract

Epstein–Barr virus (EBV) expression was investigated by immunohistochemistry (latent membrane protein 1 [LMP-1]) and in situ hybridization (EBV encoded RNA [EBER]) in biopsies from 95 patients with untreated Hodgkin’s disease (HD). Tumour EBV status was related to EBV antibody titres, spontaneous and concanavallin A induced blood lymphocyte DNA synthesis, serum levels of soluble (s) CD4, sCD8, sCD25, sCD30, sCD54, β2-microglobulin, thymidine-kinase, routine chemistry, patient characteristics, complete remission and survival. The median follow-up time was 145 months (range 60–257). Tumour EBV-positive (*n* = 30; 33%) and negative (*n* = 62; 67%) patients did not differ with regard to sex, age, stage, presence of bulky disease or B-symptoms, remission rate or survival. The proportion of EBV+ cases was significantly higher among patients with mixed cellularity histopathology (58%) as compared to the nodular sclerosis subtype (18%; *P* < 0.001). The total white blood cell (WBC) counts were significantly lower in EBV+ patients (*P* < 0.01), who also had significantly higher levels of sCD54 (*P* < 0.02) and a tendency towards lower levels of sCD30 (*P* = 0.056). Patients in the tumour EBV+ group had significantly higher IgG antibody titres to restricted early antigen (EA-R) (*P* < 0.02). Hence, clinical features and outcome were not related to tumour EBV status. However, HD patients with EBV+ tumours had elevated sCD54 levels, higher antibody titres to EA-R and decreased total WBC counts. A potential causal relationship between EBV tumour status and these findings needs to be further explored. © 1999 Cancer Research Campaign


*British Journal of Cancer* (1999) **81**(7), 1182–1187
© 1999 Cancer Research Campaign
Article no. bjoc.1999.0827

### Epstein–Barr virus expression in Hodgkin's disease in relation to patient characteristics, serum factors and blood lymphocyte function

**U Axdorph[1], A Porwit-MacDonald[2], J Sjöberg[1], G Grimfors[1], M Ekman[2], W Wang[2], P Biberfeld[2] and M Björkholm[1]**

[1]Department of Medicine, Division of Haematology Institutet and [2]Department of Pathology and Cytology, Karolinska Hospital and Institute, SE-171 76 Stockholm, Sweden

**Summary** Epstein–Barr virus (EBV) expression was investigated by immunohistochemistry (latent membrane protein 1 [LMP-1]) and in situ hybridization (EBV encoded RNA [EBER]) in biopsies from 95 patients with untreated Hodgkin's disease (HD). Tumour EBV status was related to EBV antibody titres, spontaneous and concanavallin A induced blood lymphocyte DNA synthesis, serum levels of soluble (s) CD4, sCD8, sCD25, sCD30, sCD54, β2-microglobulin, thymidine-kinase, routine chemistry, patient characteristics, complete remission and survival. The median follow-up time was 145 months (range 60–257). Tumour EBV-positive ($n$ = 30; 33%) and negative ($n$ = 62; 67%) patients did not differ with regard to sex, age, stage, presence of bulky disease or B-symptoms, remission rate or survival. The proportion of EBV+ cases was significantly higher among patients with mixed cellularity histopathology (58%) as compared to the nodular sclerosis subtype (18%; $P < 0.001$). The total white blood cell (WBC) counts were significantly lower in EBV+ patients ($P < 0.01$), who also had significantly higher levels of sCD54 ($P < 0.02$) and a tendency towards lower levels of sCD30 ($P = 0.056$). Patients in the tumour EBV+ group had significantly higher IgG antibody titres to restricted early antigen (EA-R) ($P < 0.02$). Hence, clinical features and outcome were not related to tumour EBV status. However, HD patients with EBV+ tumours had elevated sCD54 levels, higher antibody titres to EA-R and decreased total WBC counts. A potential causal relationship between EBV tumour status and these findings needs to be further explored. © 1999 Cancer Research Campaign

**Keywords:** Epstein–Barr virus; Hodgkin's disease; lymphocyte function; prognosis; sCD30; s-ICAM-1

Elevated antibody titres against certain Epstein–Barr virus (EBV) antigens have been found in individuals several years before presentation of Hodgkin's disease (HD) (Mueller et al, 1989), and in a majority of HD patients at diagnosis (Merk et al, 1992). However, a positive association between EBV antibodies and the presence of EBV in tumour cells has not been reported (Levine et al, 1994; Enblad et al, 1997). Results of recent studies show the presence of monoclonal EBV in about 30–70% of biopsies from patients with HD (Herbst et al, 1991; Lauritzen et al, 1994), and EBV has been suggested to play a role in the pathogenesis of HD. EBV-positive (EBV+) Hodgkin (H) and Reed–Sternberg (RS) cells express a restricted set of EBV gene products including latent membrane protein 1 (LMP-1) and EBV encoded RNA (EBER) (Lauritzen et al, 1994). LMP-1 is immunogenic and elicits a cellular immune response in immunocompetent individuals (Khanna et al, 1998; Murray et al, 1988). Although patients with HD usually exhibit a variable HLA class I-restricted response to EBV-derived antigens in peripheral blood lymphocytes, a marked impairment of this response within the tumours has been detected in patients with EBV+ HD tumours (Frisan et al, 1996). Of interest in this context is the observation that, in addition to oncogenic properties (Wang et al, 1985), LMP-1 also induces interleukin (IL)-10 production in Burkitt lymphoma cell lines (Nakagomi et al, 1994) and that IL-10 displays profound immunoregulatory properties and may induce a long-term antigen-specific anergic state in human CD4+ T-cells (Groux et al, 1996). It is also evident that significantly more cases with IL-10-expressing tumour cells are seen in LMP-1+ as compared to LMP-1-negative HD tumours (Herbst et al, 1996). Thus, LMP-1 may also contribute to the local inhibition of cellular immune responses observed in EBV-positive HD tumours. These observations suggest that EBV+ HD could be associated with a less favourable prognosis compared with EBV-negative HD.

In vitro, LMP-1 induces up-regulation of CD54 (intercellular adhesion molecule-1, ICAM-1) in HD cell lines (Knecht et al, 1996), but at present no increased expression of CD54 in EBV+ tumours has been reported (Kanavaros et al, 1993; Sandvej et al, 1993). To date, documentation on HD tumour cell association with EBV and its possible correlation with prognosis is limited and partly conflicting (Vestlev et al, 1992; Morente et al, 1997).

Here we have investigated the presence of EBV in HD tumour cells in relation to EBV antibody titres, blood lymphocyte function, soluble immunological factors, clinical chemistry and prognosis in a well-characterized, previously untreated cohort of patients with HD.

*Received 13 November 1998*
*Revised 21 April 1999*
*Accepted 5 May 1999*

*Correspondence to:* U Axdorph

### PATIENTS, MATERIALS AND METHODS

### Patients and biopsies (Table 1)

Ninety-five patients, diagnosed during 1974–1991 in Stockholm as having HD, were included in the study, since they had previously

**Table 1** Tumour EBV-status and clinical characteristics

| Patient category | EBV+ | EBV– | *P*-value |
|---|---|---|---|
| All patients | 30 | 62 | |
| Sex | | | |
| Male | 20 | 33 | NS |
| Female | 10 | 29 | |
| Age | | | |
| Years < 50 | 21 | 50 | NS |
| Years ≥ 50 | 9 | 12 | |
| Stage[a] | | | |
| I | 9 | 14 | |
| II | 7 | 23 | NS |
| III | 9 | 9 | |
| IV | 5 | 16 | |
| B-symptoms | | | |
| Yes | 10 | 28 | NS |
| No | 20 | 34 | |
| Bulky status | | | |
| Bulky | 5 | 17 | NS |
| Non-bulky | 25 | 45 | |
| Histopathology | | | |
| NS 1 | 6 | 28 | |
| NS 2 | 5 | 20 | 0.001 |
| MC | 19 | 14 | |
| Complete remission | | | |
| Yes | 28 | 54 | NS |
| No | 2 | 8 | |
| Relapse | | | |
| Yes | 8 | 19 | NS |
| No | 20 | 35 | |
| Prognosis | | | |
| Deceased | 10 | 21 | NS |
| Survivors | 20 | 41 | |

Fourfold table, Fischer's exact test. [a]Eight-fold table, StatXact. NS = not statistically significant.

been tested for blood lymphocyte function and various soluble serum markers (Björkholm et al, 1978; Tullgren et al, 1991; Grimfors et al, 1992; Merk et al, 1992; Axdorph et al, 1995). Three patients were discordant with regard to LMP-1 and EBER expression and were excluded from further analysis; thus 92 patients remained. The median age at diagnosis was 36 years (range 14–77 years), and the median follow-up time was 145 months (range 60–257 months) for surviving patients. The histopathology and the immunophenotyping of all biopsies were reviewed (AP) and, when necessary, complementary immunostainings for CD15, CD30, CD20 and, in addition, LN-1, CD79a, CD3, UCHL-1 and EMA were performed to confirm the diagnosis. The REAL classification (Harris, 1995) was applied including the division of nodular sclerosis (NS) according to the British National Lymphoma Investigation (MacLennan et al, 1989). There were 59 patients with NS and 33 patients with mixed cellularity (MC). Only one patient with initial lymphocyte depletion was included, this patient was reclassified to NS2. No cases of lymphocyte predominance were included in the study. The extent of disease was evaluated according to Ann Arbor staging classification (Carbone et al, 1971). Bulky disease was defined as a mediastinal mass with a diameter exceeding one-third of the maximal mediastinal width or any tumour manifestation with a diameter of >10 cm. Complete remission (CR) corresponded to complete regression of all palpable or histologically documented tumours and resolution of all radiographic and biochemical abnormalities due to HD for a minimum of 3 months.

### *Therapy*

Patients with limited disease were treated with radiotherapy, and patients in advanced stages were given chemotherapy, mainly 6–8 courses of MOPP (mechlurethamine, vincerstine sulphate, procarbazine hydrochloride and premissive) or MOPP/ABVD (doxorubicin, bleomycin, vinblastine and dacarbazine), for details see Björkholm et al (1977, 1995). All clinical and immunological studies were approved by the Ethics Committee at Karolinska Institute Institutet and all patients gave informed consent.

### *LMP-1 immunostaining*

After deparaffination and rehydration, standardized antigen retrieval was performed using microwave irradiation (Kaczorowski et al, 1994), and the slides were blocked with 5% BSA (bovine serum albumin) for 30 min. Incubation with primary antibody (mouse anti-EBV, LMP, CS 1–4; Dako A/S, Denmark) diluted in 1:40 was performed at room temperature for 1 h, followed by washings and detection of bound mouse immunoglobulin (Ig) by a double APAAP (alkaline phosphatase-anti-alkaline phosphatase) incubation (Cordell et al, 1984). The immunoreaction was visualized by detection of bound APAAP with alkaline phosphatase substrate kit I (Vector Red, Vector Laboratories, Inc., USA). Two EBV-positive index cases and B-958 cell line cytospins were used as positive controls for each batch of immunostaining. The case was considered LMP-1 or EBER-positive if any HRS cell showed a distinct membrane and/or cytoplasmic reaction.

### *EBER in situ hybridization*

In situ hybridization (ISH) was performed with FITC (fluorescein isothiocyanate)-labelled EBER probes (Dako, Glostrup, Denmark) on paraffin sections. The sections were hybridized for 2 h at 37°C in a solution with 30% formamid containing FITC-labelled probes after dewaxing, rehydration and proteinase K (10 mg $ml^{-1} \times 20$ min) digestion. For detection of the bound FITC-labelled probe, a monoclonal FITC-specific antibody and a double APAAP procedure were sequentially applied after washings and bound APAAP development with BCIP/NBT (5-bromo-4-chloro-3-indolyl phosphate/nitro blue tetrazolium). EBER+ cells were recognized by a dark blue nucleus. Parallel hybridization of a section from an EBV+ case was used as a positive control, and substitution of the probe with probe diluting solution was used as a specificity control (Wu et al, 1991).

### *Sera*

Before institution of therapy, serum samples were collected and stored at –70°C until use.

### *Blood lymphocyte stimulation*

Peripheral blood lymphocytes (PBL) were assayed for spontaneous and concanavallin A (Con A) induced DNA synthesis as previously described (Tullgren et al, 1991). Patients with an increased spontaneous (> mean of controls + 1 s.d.) and a decreased Con A (< mean of controls –1 s.d.) induced PBL DNA synthesis were defined to have an abnormal lymphocyte function.

© 1999 Cancer Research Campaign

**Table 2a** Tumour EBV-status and serum variables, all patients ($n = 92$)

| Variable | EBV+ (median, range) | EBV– (median, range) | *P*-value |
|---|---|---|---|
| ESR (mm h$^{-1}$) | 21 (2–130) | 38 (6–146) | NS |
| CRP (mg l$^{-1}$) | 9.5 (5–123) | 22 (5–164) | NS |
| Hb (g l$^{-1}$) | 134 (90–157) | 126 (83–154) | NS |
| WBC count (10$^{9}$ l$^{-1}$) | 6.9 (3.2–19.7) | 9.4 (1.9–25.7) | 0.01 |
| Eosinophil count (10$^{9}$ l$^{-1}$) | 0.15 (0–0.44) | 0.17 (0–3.00) | NS |
| Lymphocyte count (10$^{9}$ l$^{-1}$) | 1.18 (0.49–2.40) | 1.08 (0.42–3.13) | NS |
| Platelets (10$^{9}$ l$^{-1}$) | 299 (124–842) | 322 (64–705) | NS |
| IgG (g l$^{-1}$) | 12.8 (9.0–19.6) | 13.0 (3.6–27.7) | NS |
| sCD4 (U ml$^{-1}$) | 36 (10–114) | 30 (16–92) | NS |
| sCD8 (U ml$^{-1}$) | 355 (210–1260) | 380 (27–4400) | NS |
| sCD25 (U ml$^{-1}$) | 2225 (537–5132) | 1987 (317–6177) | NS |
| sCD30 (U ml$^{-1}$) | 33 (6–244) | 55 (8–1124) | NS ($P = 0.056$) |
| sCD54 (ng ml$^{-1}$) | 681 (145–1477) | 528 (110–1560) | 0.02 |
| LDH (µkat l$^{-1}$) | 7.2 (0.1–12.8) | 7.4 (3.7–16.4) | NS |

Mann–Whitney *U*-test. NS = not statistically significant.

**Table 2b** Tumour EBV-status and serum variables, patients with mixed cellularity histopathology ($n = 33$)

| | EBV+ (median, range) | EBV– (median, range) | *P*-value |
|---|---|---|---|
| ESR (mm h$^{-1}$) | 16 (2–130) | 30 (9–146) | NS ($P = 0.09$) |
| CRP (mg l$^{-1}$) | 7 (5–106) | 7 (5–147) | NS |
| Hb (g l$^{-1}$) | 137 (90–157) | 126 (90–154) | NS |
| WBC count (10$^{9}$ l$^{-1}$) | 6.6 (3.5–19.2) | 9.8 (6.9–17.2) | 0.001 |
| Eosinophil count (10$^{9}$ l$^{-1}$) | 0.12 (0–0.43) | 0.37 (0–3.00) | 0.01 |
| Lymphocyte count (10$^{9}$ l$^{-1}$) | 1.13 (0.49–2.40) | 1.37 (0.60–3.13) | NS |
| Platelets (10$^{9}$ l$^{-1}$) | 293 (124–599) | 312 (187–705) | NS |
| IgG (g l$^{-1}$) | 12.4 (9.3–19.6) | 15.6 (10.5–19.6) | 0.02 |
| sCD4 (U ml$^{-1}$) | 42 (10–114) | 40 (16–92) | NS |
| sCD8 (U ml$^{-1}$) | 430 (210–870) | 410 (190–1360) | NS |
| sCD25 (U ml$^{-1}$) | 2225 (749–4780) | 1987 (687–5317) | NS |
| sCD30 (U ml$^{-1}$) | 26 (6–79) | 130 (8–683) | 0.01 |
| sCD54 (ng ml$^{-1}$) | 672 (145–1210) | 498 (150–935) | NS ($P = 0.09$) |
| LDH (µkat l$^{-1}$) | 7.0 (4.5–9.6) | 8.8 (6–16.4) | 0.01 |

### *Serum factors*

Serum levels of sCD54 were determined by the sandwich immunoassay-method (Cellfree® ICAM-1 test kit, T-cell Diagnostics). Samples were assayed in duplicate, and mean values were calculated in compliance with the manufacturer's instruction, according to which the mean level in 66 healthy blood donors was 304 ng ml$^{-1}$ with an upper limit of 460 ng ml$^{-1}$ (mean + 2 s.d.). sCD4 (Cellfree CD4, T-cell Sciences), sCD8 (Cellfree T8, T-cell Sciences), sCD25 (Cellfree IL-2R test kit, T-cell Diagnostics) and sCD30 (Dako CD30 [Ki-1 Antigen] enzyme-linked immunosorbent assay (ELISA) were determined in similar ways by sandwich immunoassay methods.

β2-Microglobulin (β2-MG) was analysed by a competitive luminometric assay (LIA-mat® β2-Microglobulin, Cambridge Life Sciences plc, UK). Thymidine-kinase (TK) was measured in a radio-enzyme assay (Prolifigen® TK-REA, AB Sangtec Medical).

Erythrocyte sedimentation rate (ESR), C-reactive protein (CRP), haemoglobin, white blood cell (WBC) counts, platelets, serum orosomucoid, haptoglobin, albumin, IgG, IgA, IgM and lactate dehydrogenase (LDH) were determined according to standard methods.

### *Serum EBV antibodies*

IgG antibodies to viral capsid antigen (VCA) and to early antigen diffuse (EA-D) and early antigen restricted (EA-R) were detected by indirect immunofluorescence according to Henle et al (1974). Antibodies to Epstein–Barr nuclear antigen (EBNA) were measured by anticomplement indirect immunofluoroscence staining (ACIF) using Raji cells as described by Reedman and Klein (1973). Detectable titres were defined as IgG VCA ≥1:20, IgG EA-D, IgG EA-R and EBNA ≥ 1:5 (Enblad et al, 1997).

### Statistical methods

Statistica version 4.1 program was used. Significance of differences between groups was tested by Mann–Whitney *U*-test. Fischer's exact test was used in fourfold tables (Armitage and Berry, 1987) and StatXact was used in eightfold tables (Mehta et al, 1984). In analyses regarding geometric mean titres (GMT), the titres were regarded as approximately logarithmic-normally distributed, why the titres were logarithmically transformed. These mean values were tested by Student's *t*-test. Survival analyses were performed by Kaplan–Meier curves and the log-rank test.

 © 1999 Cancer Research Campaign

**Table 3** Tumour EBV-status and EBV antibody titres (geometric mean)

| | EBV+ | EBV– | *P*-value |
|---|---|---|---|
| IgG VCA | *n* = 15<br>485.1<br>(255.5–920.0) | *n* = 32<br>406.1<br>(268.6–613.9) | NS |
| EBNA | *n* = 14<br>51.2<br>(20.3–129.6) | *n* = 27<br>63.5<br>(34.1–118.1) | NS |
| IgG EA-D | *n* = 10<br>13.2<br>(8.2–21.2) | *n* = 22<br>11.7<br>(8.4–16.2) | NS |
| IgG EA-R | *n* = 9<br>29.4<br>(10.3–83.8) | *n* = 23<br>12.7<br>(10.7–15.1) | 0.02 |

Student's *t*-test, 95% confidence interval. NS = not statistically significant.

Overall survival was defined as the time from diagnosis to death of any cause. Cause-specific survival was defined as the time from diagnosis to death from HD or death related to HD (cardiovascular second malignancy). Observations were censored by end of follow-up or death without signs of HD.

### RESULTS

The results of the various assays are presented in Tables 1–3. The proportion of tumour EBV+ cases was significantly higher among patients with MC (58%) histopathology as compared to the NS subtype (18%; $P < 0.001$). The EBV+ and EBV– patients did not differ significantly with regard to age, sex, stage, presence of bulky disease or B-symptoms, remission rate or prognosis (Table 1). Cause-specific or overall survival did not differ significantly between EBV+ and EBV– patients (data not shown). No significant differences were seen even when the analyses were restricted to the MC-group (data not shown).

Total WBC counts were significantly lower among the EBV+ patients ($P < 0.01$, Table 2). No differences were found in total lymphocyte or eosinophil WBC counts between the EBV+ and EBV– patients. Levels of ESR and CRP were slightly lower in the EBV+ group, but the difference did not reach statistical significance (Table 2). No significant differences between the EBV+ and EBV– patients were seen in serum orosomucoid, haptoglobin, albumin, IgA and IgM, β2-MG and TK (data not shown), haemoglobin, platelets, IgG and LDH (Table 2). Serum levels of IgG were significantly lower in EBV+ than EBV– patients with subtype MC ($P < 0.02$), as were the total ($P < 0.001$) and eosinophil ($P < 0.01$) WBC counts and LDH ($P < 0.01$, Table 2).

The levels of sCD54 (sICAM-1) were significantly higher in the EBV+ group ($P < 0.02$, Table 2). Levels of sCD30 (Ki-1) had a tendency to be lower among the EBV+ patients, but the difference did not reach statistical significance ($P = 0.056$, Table 2). When this analysis was restricted to the MC-group, the levels of sCD30 were significantly lower in the EBV+ group ($P < 0.01$, Table 2).

No correlation between spontaneous blood lymphocyte DNA synthesis or Con A induced blood lymphocyte DNA synthesis and EBV-status of the patients was seen (data not shown). Thus, lymphocyte abnormalities did not differ significantly between EBV+ and EBV– patients (data not shown).

EBV antibodies (IgG VCA and EBNA titres) were detectable in all of the 47 tested patients as evidence of a previous EBV infection. Thirty-two patients with detectable IgG VCA (68%) had EBV– biopsies. IgG VCA, IgG EA-D and EBNA titres did not differ between patients with EBV+ and negative biopsies respectively. However, significantly higher IgG EA-R titres were seen in patients with EBV+ biopsies ($P < 0.02$, Table 3).

### DISCUSSION

In this series of patients with HD, the proportion of EBV+ tumour biopsies was 33%, with the majority (63%) diagnosed as MC cases, which confirms previous findings (Herbst et al, 1991; Pallesen et al, 1991; Pinkus et al, 1994; Enblad et al, 1997). This is the first report to describe an association between a positive tumour EBV status and increased sCD54 levels, elevated titres of EA-R and a decreased WBC count in untreated HD. The increased concentration of sCD54 in EBV+ patients may reflect the in vitro finding that LMP-1 induces B-cell expression of CD54 (Wang et al, 1988), which is the ligand for lymphocyte function-associated antigen 1 (LFA-1) (Marlin and Springer, 1987). ICAM-1/LFA-1 regulates cell–stromal and cell–cell interactions such as leucocyte adhesion (Makgoba et al, 1988*a*), mitogen-induced T-cell proliferation (Dougherty et al, 1988) and T-cell-mediated cytotoxicity (Makgoba et al, 1988*b*). Furthermore, the CD30 ligand (CD30L), a member of the tumour necrosis factor/nerve growth factor (TNF/NGF) superfamily is known to up-regulate CD54 expression by CD30+ cultured HRS cells, and to increase shedding of surface-bound CD54 (Gruss et al, 1996). Pizzolo et al (1993) have also reported that an increased expression of CD54 in HD tissue was associated with high levels of serum sCD54, and with more advanced clinical stage and the presence of constitutional symptoms and bulky disease. In a previous study, elevated sCD54 values were observed in patients with high age, advanced disease, constitutional symptoms, lymphocytic depletion histopathology, decreased remission rate and 5-year survival and abnormal lymphocyte function (Axdorph et al, 1995). In contrast, other authors did not find any difference in expression of CD54 between LMP-1+ and LMP-1– tumour biopsies from HD patients. However, the numbers of patients included in these studies were quite limited (Sandvej et al, 1992; Kanavaros et al, 1993). Our results showed EBV+ patients to have significantly higher levels of sCD54 ($P < 0.02$) suggesting that indeed there is an association between sCD54 and LMP-1 in HD tumours.

CD30 is a member of the TNF/NGF receptor superfamily usually expressed on activated lymphocytes. A correlation between the expression of LMP-1 and the detection of CD30 in HRS cells has been suggested (Kanavaros et al, 1993). The CD30 antigen has also been considered as a marker for HRS cells (Gruss and Hermann, 1996). Moreover, the soluble form of CD30 (sCD30) has been shown to be an indicator of disease activity and a predictor of prognosis (Nadali et al, 1994), which is in good agreement with our own unpublished observations. The CD30L is expressed on activated T-cells and has the ability to induce apoptosis in several CD30+ cell lines (Younes et al, 1997). Thus, the increase in sCD30 can decrease the availability of CD30L on peripheral blood effector lymphocytes. This blocking of CD30L apoptosis-inducing activity may in turn impair immunosurveillance and contribute to the poor prognosis observed in patients with elevated sCD30 levels (Younes et al, 1997). In the present study, no strong correlation between tumour EBV status and sCD30 levels was observed; EBV+ patients with MC histopathology status had significantly lower sCD30 values ($P < 0.01$) than the corresponding EBV– group. In addition,

© 1999 Cancer Research Campaign 

cause-specific and overall survival was the same in these two patient groups respectively.

As previously shown, increased spontaneous and decreased Con A induced blood lymphocyte DNA synthesis both predict a poor prognosis in HD (Björkholm et al, 1978; Wedelin et al, 1982; Tullgren et al, 1991). In addition, increased levels of sCD8 was associated with lymphocyte abnormalities and a dismal prognosis (Grimfors et al, 1992). In this study no association was found between tumour EBV status and spontaneous or Con A induced blood lymphocyte DNA synthesis or sCD8 levels. Evidently, an abnormal lymphocyte function can not directly be correlated to the EBV-status of the tumour.

The findings of significantly lower total WBC and eosinophil cell counts ($P < 0.001$ and $P < 0.01$ respectively) and IgG ($P < 0.02$), in the subgroup of EBV+ MC patients might possibly indicate a down-modulation of the inflammatory response to the tumour, which is supported by the tendency ($P = 0.09$), towards lower ESR values in this patient group.

The relationship between the tumour EBV status of HD patients and the serological responses has recently been analysed. No correlation between serum antibody pattern and EBV tumour status was found by Levine et al (1994) or Enblad et al (1997). Previous reports have described EBV replication in RS cells as an exceptional event (Brousset et al, 1993; Bibeau et al, 1994). Pallesen et al (1991) showed that, in general, activation of EBV early genes occurs only infrequently in RS cells. Enblad et al (1997) found a significantly lower proportion of patients with detectable titres of IgG EA-R in the tumour EBV+ patients in comparison to EBV–, but in that study, no significant difference in EA-R antibody titres was recorded between EBV+ and EBV– patients. In the present study, IgG VCA, IgG EA-D and EBNA titres did not differ between tumour EBV+ and EBV– patients, while the EA-R antibody titre was significantly higher in the EBV+ group ($P < 0.02$, Table 3), suggesting the presence of EBV replication. The discrepancy, if any, between the results reported by Enblad et al and the present series, may be due to differences in the patient populations.

Few studies have related tumour EBV status to patient outcome. In addition, our findings are in agreement with reports of Vestlev et al (1992) and Enblad et al (1997), who found no difference in progression-free or cause-specific survival respectively, between EBV+ and EBV– patients, but disagree with the results reported by Morente et al (1997), where patients with EBV+ biopsies had a longer overall survival. However, there was a tendency to more relapses among EBV– patients in our series.

In summary, in our patient series a higher proportion of EBV+ tumour biopsies was confirmed in patients with MC histopathology. Despite the potential pathogenetic role of EBV in HD, there was no association between tumour EBV status and lymphocyte function or outcome. A potential causal relationship between EBV tumour status and sCD54 and total WBC counts needs to be further explored.

### ACKNOWLEDGEMENTS

This study was supported by grants from the Swedish Cancer Society and Karolinska Institute Foundations.

### REFERENCES

Armitage P and Berry G (1987) *Statistical Methods in Medical Research*, 2nd edn, pp. 125–132. Blackwell, Cambridge

Axdorph U, Grimfors G, Landgren O, Giscombe R, Johansson B and Björkholm M (1995) Serum levels of sICAM-1 are correlated to tumour burden and blood lymphocyte functions in untreated Hodgkin's disease. Third International Symposium on Hodgkin's Lymphoma, Köln (abstract)

Bibeau F, Brousset P, Knecht H, Meggetto F, Drouet E, Rubin B and Delsol G (1994) Epstein–Barr virus replication in Hodgkin's disease. *Bull Cancer* **81**: 114–118

Björkholm M, Holm G, Mellstedt H, Johansson B and Askergren J (1977) Prognostic factors in Hodgkin's disease. I. Analysis of histopathology, stage distribution and results of therapy. *Scand J Haematol* **19**: 487–495

Björkholm M, Holm G, Mellstedt H, Johansson B, Killander D, Sundblad R and Söderberg G (1978) Prognostic factors in Hodgkin's disease. II. The role of lymphocyte defect. *Scand J Haematol* **20**: 306–308

Björkholm M, Axdorph U, Grimfors G, Merk K, Johansson B, Svedmyr E, Mellstedt H and Holm G (1995) Fixed versus response adapted MOPP/ABVD chemotherapy in Hodgkin's disease. *Ann Oncol* **6**: 895–899

Brousset P, Knecht H, Rubin B, Drouet E, Chittal S, Meggetto F, Saati TA, Bachmann E, Denoyel G, Sergeant A and Delsol G (1993) Demonstration of Epstein-Barr virus replication in Reed-Sternberg cells of Hodgkin's disease. *Blood* **82**: 872–876

Carbone PP, Kaplan HS, Musshoff K, Smithers DW and Tubiana M (1971) Report of the Committee on Hodgkin's disease staging classification. *Cancer Res* **31**: 1860–1861

Cordell JL, Falini B, Erber WN, Ghosh AK, Abdulaziz Z, MacDonald S, Pulford KA, Stein H and Mason DY (1984) Immunoenzymatic labeling of monoclonal antibodies using immune complexes of alkaline phosphatase and monoclonal anti-alkaline phosphatase (APAAP complexes). *J Histochem Cytochem* **32**: 219

Dougherty G, Murdoch S and Hogg N (1988) The function of human intercellular adhesion molecule-1 (ICAM-1) in the generation of an immune response. *Eur J Immunol* **18**: 35–39

Enblad G, Sandvej K, Lennette E, Sundström C, Klein G, Glimelius B and Pallesen G (1997) Lack of correlation between EBV serology and presence of EBV in the Hodgkin and Reed–Sternberg cells of patients with Hodgkin's disease. *Int J Cancer* **72**: 394–397

Frisan T, Sjöberg J, Dolcetti R, Boiocchi M, de Re V, Carbone A, Brautbar C, Battat S, Biberfeld P, Ekman M, Christensson B, Sundström C, Björkholm M, Pisa P and Masucci MG (1995) Local suppression of Epstein-Barr virus (EBV)-specific cytotoxicity in biopsies of EBV-positive Hodgkin's disease. *Blood* **86**: 1493–1501

Grimfors G, Andersson B, Tullgren O, Giscombe R, Holm G, Johansson B and Björkholm M (1992) Increased serum CD 8 soluble antigen level is associated with blood lymphocyte abnormalities and other established indicators of poor prognosis in adult Hodgkin's disease. *Br J Haematol* **80**: 166–171

Groux H, Bigler M, de Vries JE and Roncarolo MG (1996) Interleukin-10 induces a long-term antigen-specific anergic state in human CD4+ T cells. *J Exp Med* **184**: 19–29

Gruss HJ and Hermann F (1996) CD30 ligand, a member of the TNF ligand superfamily, with growth and activation control for CD30+ lymphoid and lymphoma cells. *Leukemia and Lymphoma* **20**: 397–409

Gruss HJ, Scheffran I, Hubinger G, Duyster J and Herrman F (1996) The CD30 ligand and CD40 ligand regulate CD54 surface expression and release of its soluble form by cultured Hodgkin and Reed–Sternberg cells. *Leukemia* **10**: 829–835

Harris LN (1995) A practical approach to the pathology of lymphoid neoplasms: a revised European–American classification from the international lymphoma study group. In: *Important Advances in Oncology*, De Vita, Hellman and Rosenberg (eds), pp. 111–140. JB Lippincott: Philadelphia

Henle W, Henle GE and Horwitz CA (1974) Epstein-Barr virus specific diagnostic tests in infectious mononucleosis. *Hum Pathol* **5**: 551–565

Herbst H, Dallenbach F, Hummel M, Niedobitek G, Pileri S, Muller-Lantsch N and Stein H (1991) Epstein-Barr virus latent membrane protein expression in Hodgkin and Reed-Sternberg cells. *Proc Natl Acad Sci USA* **88**: 4766–4770

Herbst H, Foss HD, Samol J, Araujo I, Klotzbach H, Krause H, Agathanggelou A, Niedobitek G and Stein H (1996) Frequent expression of interleukin-10 in Epstein-Barr virus-harboring tumour cells of Hodgkin's disease. *Blood* **87**: 2918–2929

Kaczorowski S, Kaczorowska M and Christensson B (1994) Expression of EBV encoded latent membrane protein 1 (LMP-1) and bcl-2 protein in childhood and adult Hodgkin's disease: application of microwave irradiation for antigen retrival. *Leukemia Lymphoma* **13**: 273–283

Kanavaros P, Jiwa M, van der Valk P, Walboomers J, Horstman A and Meijer C (1993) Expression of Epstein-Barr virus latent gene products and related cellular activation and adhesion molecules in Hodgkin's disease and non-Hodgkin's lymphomas arising in patients without overt pre-existing immunodeficiency. *Hum Pathol* **24**: 725–729

 *© 1999 Cancer Research Campaign*

Khanna R, Burrows SR, Nicholls J and Poulsen LM (1998) Identification of cytotoxic T cell epitopes within Epstein-Barr virus (EBV) oncogene latent membrane protein 1 (LMP 1): evidence for HLA A2 supertype-restricted immune recognition of EBV-infected cells by LMP 1-specific cytotoxic T lymphocytes. *Eur J Immunol* **28**: 451–458

Knecht H, McQuain C, Martin J, Rothenberger S, Drexler HG, Berger C, Bachmann E, Kittler ELW, Odermatt BF and Quesenberry PJ (1996) Expression of the LMP1 oncoprotein in the EBV-negative Hodgkin's disease cell line L-428 is associated with Reed-Sternberg cell morphology. *Oncogene* **13**: 947–953

Lauritzen AF, Hörding U and Nielsen HW (1994) Epstein-Barr virus and Hodgkin's disease: a comparative immunological, in situ hybridization, and polymerase chain reaction study. *APMIS* **102**: 495–500

Levine PH, Pallesen G, Ebbesen P, Harris N, Evans AS and Mueller N (1994) Evaluation of Epstein-Barr virus antibody patterns and detection of viral markers in the biopsies of patients with Hodgkin's disease. *Int J Cancer* **59**: 48–50

MacLennan KA, Bennett MH, Tu A, Vaughan Hudson B, Easterling J, Vaughan Hudson G and Jelliffe AM (1989) Relationship of histopathologic features to survival and relapse in nodular sclerosing Hodgkin's disease. *Cancer* **64**: 1686–1693

Makgoba MW, Sanders ME, Ginther Luce GE, Dustin ML, Springer TA, Clark EA, Mannoni P and Shaw S (1988*a*) ICAM-1 a ligand for LFA-1-dependent adhesion of B, T and myeloid cells. *Nature* **331**: 86–88

Makgoba MW, Sanders ME, Ginther Luce GE, Gugel EA, Dustin ML, Springer TA and Shaw S (1988*b*) Functional evidence that intercellular adhesion molecule-1 (ICAM-1) is a ligand for LFA-1-dependent adhesion in T cell-mediated cytotoxicity. *Eur J Immunol* **18**: 637–640

Marlin SD and Springer TA (1987) Purified intercellular adhesion molecule-1 (ICAM-1) is a ligand for lymphocyte function-associated antigen 1 (LFA-1). *Cell* **51**: 813–819

Mehta CR, Patel NR and Tsiatis AA (1984) Exact significance testing to establish treatment equivalence for ordered categorial data. *Biometrics* **40**: 819–825

Merk K, Lennette E, Holm G, Johansson B, Klein G and Björkholm M (1992) Antibodies to Epstein-Barr virus in relation to clinical characteristics of untreated patients with Hodgkin's disease (paper 5). In: *An Epidemiological Approach to the Etiology of Hodgkin's Disease*. Thesis. Karl Merk, Department of Oncology, Karolinska Institute, Stockholm

Morente MM, Piris MA, Abraira V, Acevedo A, Aguilera B, Bellas C, Fraga M, Garcia-Del-Moral R, Gomez-Marcos F, Menarguez J, Oliva H, Sanchez-Beato M and Montalban C (1997) Adverse clinical outcome in Hodgkin's disease is associated with loss of retinoblastoma protein expression, high Ki-67 proliferation index, and absence of Epstein-Barr virus-latent membrane protein 1 expression. *Blood* **90**: 2429–2436

Mueller N, Evans A, Harris NL, Comstock GW, Jellum E, Magnus K, Orentreich N, Polk F and Vogelman J (1989) Hodgkin's disease and Epstein-Barr virus altered antibody pattern before diagnosis. *N Engl J Med* **320**: 689–695

Murray RJ, Wang D, Young LS, Wang F, Rowe M, Kieff E and Rickinson AB (1988) Epstein-Barr virus-specific cytotoxic T-cell recognition of transfectants expressing the virus-coded latent membrane protein LMP. *J Virol* **62**: 3747–3755

Nadali G, Vinante F, Ambrosetti A, Todeschini G, Veneri D, Zanotti R, Meneghini V, Ricetti MM, Benedetti F, Vassanelli A, Perona G, Chilosi M, Menestrina F, Fiacchini M, Stein H and Pizzolo G (1994) Serum levels of soluble CD30 are elevated in the majority of untreated patients with Hodgkin's disease and correlate with clinical features and prognosis. *J Clin Oncol* **12**: 793–797

Nakagomi H, Dolcetti R, Bejarano MT, Pisa P, Kiessling R and Masucci MG (1994) The Epstein-Barr virus latent membrane protein-1 (LMP-1) induces interleukin 10 production in Burkitt lymphoma lines. *Int J Cancer* **57**: 240–244

Pallesen G, Hamilton-Dutoit JS, Rowe M and Young LS (1991) Expression of Epstein-Barr virus (EBV) latent gene products in tumour cells of Hodgkin's disease. *Lancet* **337**: 320–322

Pinkus GS, Lones M, Shintaku P and Sad JW (1994) Immunohistochemical detection of Epstein-Barr virus-encoded latent membrane protein in Reed–Sternberg cells and variants of Hodgkin's disease. *Modern Pathol* **7**: 454–461

Pizzolo G, Vinante F, Nadali G, Ricetti MM, Morosato L, Marrocchella R, Vincenzi C, Semenzato G and Chilosi M (1993) ICAM-1 tissue overexpression associated with increased serum levels of its soluble form in Hodgkin's disease. *Br J Haematol* **84**: 161–162

Reedman BM and Klein G (1973) Cellular localization of an Epstein-Barr virus (EBV)-associated complement-fixing antigen in producer and non-producer lymphoblastoid cell lines. *Int J Cancer* **11**: 499–520

Sandvej KB, Hamilton-Dutoit SJ and Pallesen G (1993) Influence of Epstein-Barr virus encoded latent membrane protein 1 on the expression of CD23 antigen, CD54 and LFA-3 in Hodgkin and Reed–Sternberg cells. A morphometric analysis. *Leukemia Lymphoma* **9**: 95–101

Tullgren O, Grimfors G, Holm G, Johansson B, Svedmyr E, Wedelin C, Mellstedt H, Merk K and Björkholm M (1991) Lymphocyte abnormalities predicting a poor prognosis in Hodgkin's disease. *Cancer* **68**: 768–775

Vestlev PM, Pallesen G, Sandvej K, Hamilton-Dutoit SJ and Bendtzen SM (1992) Prognosis of Hodgkin's disease is not influenced by Epstein-Barr virus latent protein. *Int J Cancer* **50**: 670–671

Wang D, Liebowitz D and Kieff E (1985) An EBV membrane protein expressed in immortalized lymphocytes transforms established rodent cells. *Cell* **43**: 831–840

Wang D, Liebowitz D, Wang F, Gregory C, Rickinson A, Larson R, Springer T and Kieff E (1988) Epstein-Barr virus latent infection membrane protein alters the human B-lymphocyte phenotype: deletion of the amino terminus abolishes activity. *J Virol* **62**: 4173–4184

Wedelin C, Björkholm M, Holm G, Ogenstad S, Johansson B and Mellstedt H (1982) Lymphocyte function in untreated Hodgkin's disease: an important predictor of prognosis. *Br J Cancer* **45**: 70–79

Wu TC, Mann RB, Epstein JI, MacMahon E, Lee WA, Charache P, Hayward SD, Kurman RJ, Hayward GS and Ambinder RF (1991) Abundant expression of EBER1 small nuclear RNA in nasopharyngeal carcinoma. *Am J Pathol* **138**: 1461–1469

Younes A, Consoli U, Snell V, Clodi K, Kliche KO, Palmer JL, Gruss HJ, Armitage R, Thomas EK, Cabanillas F and Andreeff M (1997) CD30 ligand in lymphoma patients with CD30+ tumours. *J Clin Oncol* **15**: 3355–3362

*© 1999 Cancer Research Campaign*
